# Do Infield Softball Masks Effectively Reduce Facial Fracture Risk?

**DOI:** 10.1007/s10439-018-02144-6

**Published:** 2018-10-25

**Authors:** Tyler P. Morris, Ryan A. Gellner, Steven Rowson

**Affiliations:** Virginia Polytechnic Institute and State University, Blacksburg, VA USA

**Keywords:** Head impacts, Ball impact, Injury, Biomechanics, Zygoma, Maxilla, Orbital

## Abstract

Infield softball masks are intended to reduce facial fracture risk, but are rarely worn. The objective of this study was to evaluate the effectiveness of infield masks’ ability to attenuate facial fracture risk over a range of designs and materials. To simulate batted ball impacts, a customized pitching machine was used to propel softballs at 24.6 ± 0.51 m/s. The balls impacted locations centered over the maxilla and zygoma bones of a FOCUS headform. The FOCUS headform was attached to a 50th percentile Hybrid III neck and secured to a slider table. Facial fracture risk of each facial bone was compared between masks and impact locations using peak resultant forces. Analysis of these data showed that the mask material and the distance between the mask and the impacted facial bone were key factors in determining a mask’s performance. The effectiveness of masks varied. It was found that a metal mask with a separation distance ≥ 35 mm away from the maxilla and ≥ 25 mm away from the zygoma best reduced facial fracture risk for these test configurations. Plastic masks performed worse because they excessively deformed allowing ball contact with the face. This study assesses various mask designs for their ability to reduce facial fracture and suggests design recommendations based on the impact configurations tested.

## Introduction

Approximately 41 million people participate in recreational softball annually, as reported by the American Softball Association.^12^ Compared to baseball, softball has a greater overall injury rate, especially because the injury rate while fielding batted balls is higher.^10^ This is likely due to the difference in field size between baseball and softball. Softball fields are smaller than baseball fields. For comparison, a regulation baseball field’s pitching mound is 60.5 feet away from home plate and there are 90 feet in between each base, while a softball field’s pitching mound is only 43 feet away from home plate with 60 feet in between the bases. The reduction in field size decreases the amount of time fielders have to react to a batted ball and increases their chances of sustaining an injury due to ball impact. Ball impact is one of the leading causes of injury in softball and the most frequent cause of facial fracture.^15^ Of the fractures caused from ball impacts, 42% of them were due to a batted ball.^1^,^18^ The highest percentage of facial fractures in softball occur in the midface region (zygoma, orbital, nasal, and maxilla), so this region was the area of interest for this study.^1^,^21^

Facial fracture risk is dependent on the stress enacted on the underlying bone. These stresses are caused by forces transmitted from blunt impacts. Studies done by Cormier *et al*. established force thresholds for facial bone fracture. These thresholds were established by instrumenting cadaver heads with acoustic emission sensors, then loading the facial bones with a flat-faced cylindrical impactor. Acoustic emissions sensors were used to determine fracture force because the peak force is not always the fracture force in facial bones. Using the censored data collected, fracture risk curves of each facial bone were developed for both direct and lateral impacts.^4^,^6^,^13^

The United States Consumer Product Safety Commission (CPSC) reported that 36% of all baseball and softball injuries could be prevented, or reduced in severity with the use of safety equipment.^1^ Since 2006, it has been a requirement that all softball batters wear a batting helmet with a facemask, however, infielder masks are still not required in the sport. Although batting helmet facemasks are effective at reducing facial injury in both baseball and softball, estimating a prevention of 3900 facial injuries annually, 62% of the players struck by a batted ball were in a fielders position that would not be wearing a batter’s helmet.^1^,^17^,^18^ It is difficult for high school and collegiate leagues to mandate the use of infielder masks because the National Operating Committee on Standards for Athletic Equipment (NOCSAE) does not certify them. NOCSAE has not developed a standard for a facemask only device because mask-only headgear does not provide enough protection to prevent head injury. In order for NOCSAE to certify a mask of this type, it would have to be heavily padded and have a shell that encompasses the head, similar to a catcher’s mask.^16^

There is a lot of discussion about requiring infield masks in softball, but there is still a lack of data on the effectiveness of these masks to support these discussions. To our knowledge, no prior studies or information is available in the literature on how effective infield softball masks are at reducing facial fracture risk or head accelerations. Even though there have been no studies looking into the effectiveness of infield softball masks, there have been studies conducted to analyze the performance of baseball catcher’s masks.^2^,^11^,^14^,^19^,^20^ These studies have shown that there are differences in performance based on the design and material of a catcher’s mask and by wearing a catcher’s mask, head accelerations are reduced by approximately 85% in comparison to a bare headform impact.^2^,^11^,^14^,^19^,^20^ Although these studies were focused on catcher’s masks, most of the impacts were directed at the facemask, showing promise that a mask only device is capable of reducing facial fracture and head injury risk. In addition to these studies, new helmet add-ons like the C-flap have been making an appearance in major league baseball (MLB) in an effort to prevent facial fracture while batting; which has further increased the awareness of facial fracture as an issue in the sport. The objective of this study was to evaluate the effectiveness of infielder masks’ ability to attenuate facial fracture risk. A better understanding of these masks’ performances will help determine the benefits of wearing an infielder’s mask and aid in optimizing mask design.

## Methods

To simulate batted ball impacts into a softball fielder’s face, softballs were projected using a softball pitching machine. The dual wheeled, electric motor-driven machine (Jugs Sports Combination Pitching Machine Model SR3616-681-7, Tualatin, OR) was customized and anchored to the floor to reduce unwanted vibration. Each wheel had an independent speed dial with digits ranging from 0 to 100 that could be set to acquire the desired speed. The wheels were pressurized to 17 psi and their speed dials maintained at least a 35 digit offset from one another to prevent the softball from knuckling, as specified in the manual. In order to minimize potential differences in softball positioning during loading, custom ball holders were constructed to create a homogeneous loading orientation for all impacts. The softball velocity was calculated over the final 10.16 cm of the softball mount using a dual laser velocity gate sensor (Velocity Timer Model 1204, KME Company, Troy, MI). The pitching machine was able to yield the desired impact velocity of 24.6 ± 0.51 m/s, which resembled the average batted ball velocity of a female high school softball player.^1^ The customized pitching machine possessed an impact location accuracy within a 0.635 cm radius circle, which was verified from a previous study using the same machine.^2^ Only line drives were replicated in this study because there was no literature to indicate that these impact events occur more frequently when fielding a line drive, or a bouncing ball. Line drive impacts have known boundary conditions, which is why they were chosen to be recreated in the laboratory.^1^ Bouncing balls possess numerous rebound velocities and bounce angles that have yet to be quantified in the literature. All the impacts modeled were direct frontal impacts that are thought to be most common, not oblique. Previous research has suggested that there is little difference in catcher’s masks reducing head accelerations based on ball trajectory.^20^

The softballs used to test the infield masks were 12 in. in circumference, weighed 7.0 oz, and were manufactured by Rawlings (model C12RYLAH). Metal masks evaluated included the All-Star: Vela, Bangerz: HS-6500, Champro: The Grill, Rawlings: Fielders Mask, Schutt: Fielders Guard, and Schutt: Titanium Fielders Guard. Plastic masks evaluated included the Defender Sports: Defender Sports Shield and Markwort: Game Face (large and medium). Each of these masks were equipped with adjustable head straps that allowed for proper sizing. In addition, all the masks possessed some type of foam padding that lined the forehead contact area. All masks except the Bangerz were equipped with some type of chin pad, however they were comprised of various materials. The Bangerz mask was the only mask that had foam lining the entire frame of the mask, but only protected the nasal to forehead region, leaving the facial area below the nose exposed. These infielder masks were chosen because they appeared to represent a range of designs and materials that have been commonly used for infielder masks on the market.

Facial fracture risk for each infield mask was evaluated using the response of a surrogate headform. A Facial and Ocular CountermeasUre for Safety (FOCUS) headform and a 50th percentile male Hybrid III neck were affixed to a 16 kg sliding table that mimicked the inertial properties of the upper torso. The FOCUS headform is equipped with ten tri-axial titanium force plates that measure the loading patterns of the eyes and facial bones. The facial bones modeled in the FOCUS headform are the right and left frontal bone, the right and left zygoma, the right and left maxilla, the nasal bone, and the mandible.^3^,^9^ For this study, the eye data were excluded and only the facial bone data were collected. Data acquisition was conducted using a TDAS Slice Pro (DTS, Seal Beach, CA) system with a sampling rate of 20 kHz. The amount of data taken for each impact was 150 ms (50 ms before impact and 100 ms after impact). The data acquisition system was triggered using a threshold of 5 g at the center of gravity of the headform in the direction of the impact.

**Figure 1:**
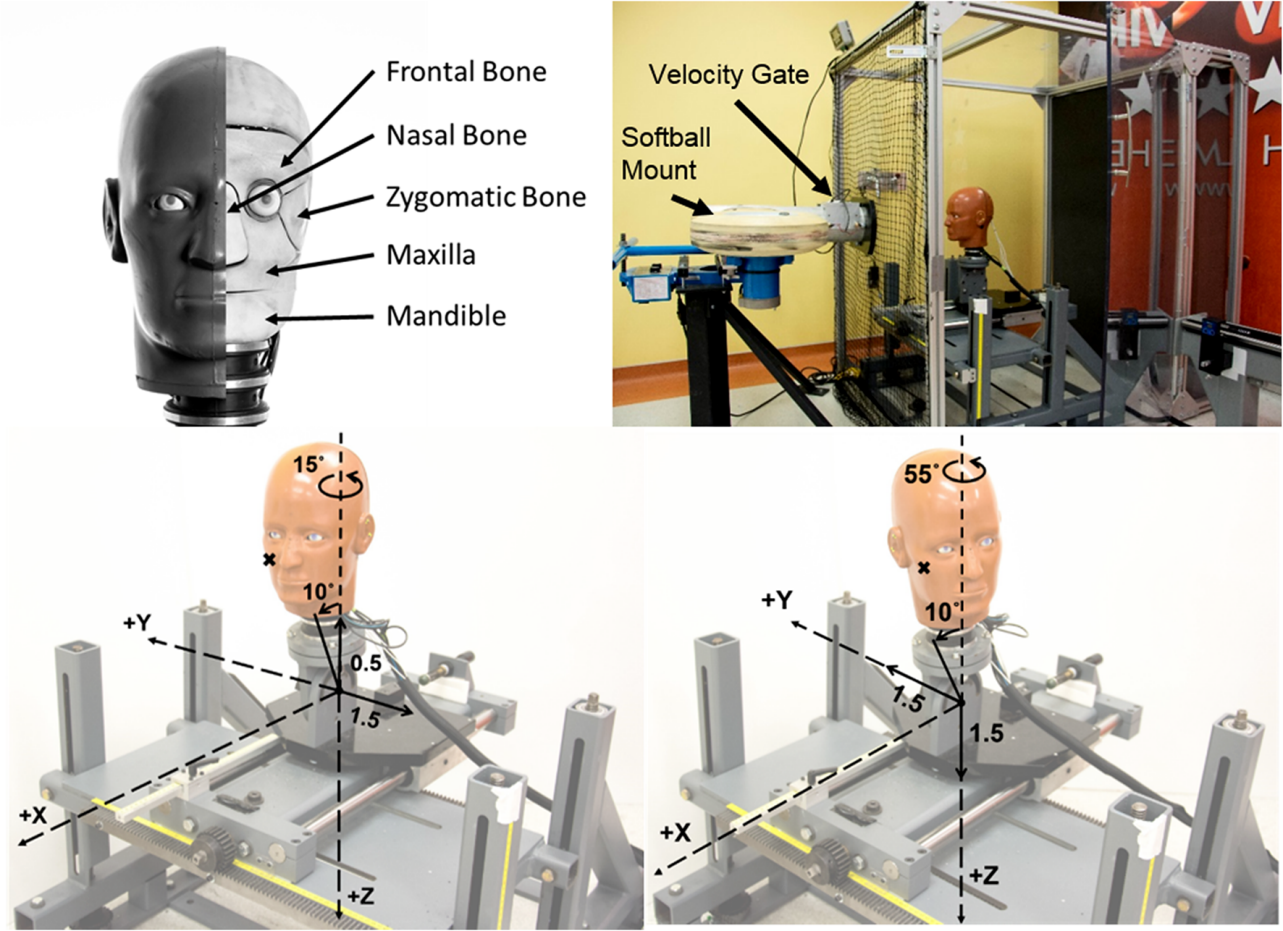
Top left: exposed FOCUS headform showing the underlying facial bones. Top right: anchored customized pitching machine that projects softballs into a FOCUS headform attached to a 50th percentile male Hybrid III neck that is mounted to a 16-kg sliding table. Bottom left: maxilla impact location (M) with translations and rotations from the reference location. Bottom right: zygoma impact location (Z) with translations and rotations from the reference location.

The two locations chosen for impact testing were centered over the maxilla (location M) and the zygoma (location Z) of the headform. The maxilla bone was selected because it was a commonly fractured bone in the midface region and the zygoma bone was selected because it was the most prevalent of all facial fractures.^1^,^15^,^21^ Locations are referenced from a zero location on the FOCUS headform. The zero location is when the FOCUS headform is centered in front of the launcher with no rotations about any axis, and positioned so the middle of the muzzle is located at the tip of the nose. For both locations the headform was positioned 36.5 cm away from the end of the launcher in the *x* direction using the SAE J211 coordinate system of the head. The headform was also tilted 10° toward the launcher in order to replicate the natural infielder stance prior to the ball being batted. This stance is when a player is slightly crouched, with their head up, and leaning forward onto their toes in anticipation for the ball. For the maxilla impact location the *y* and *z* translations on the slider table from the zero location were − 1.5 and − 0.5 cm respectively and the headform was rotated − 15° about the *z* axis. For the zygoma impact location, the *y* and *z* translations from the zero location were 1.5 and 1.5 cm respectively and the head was rotated − 55° about the *z*-axis. These translations and rotations correspond with the SAE J211 coordinate system of the head. The facial bones of the FOCUS, experimental setup, and impact locations are depicted in Fig. 1.

The masks were tested at an impact speed equivalent to the average batted ball speed of female high school softball players, 24.6 ± 0.51 m/s.^1^ This speed corresponded to the right wheel set to 67 on the speed dial and the left wheel set to 27 on the speed dial. Four masks of each type were tested. Two of the four masks were used for the maxilla location (M), while the other two were used to test the zygoma location (Z). Each mask was only impacted once because of deformation upon impact, totaling two trials at each location and a new ball was used for each mask model. Because there were no fitting directions from the manufacturers, each mask was positioned on the head using best judgment. The chin pad was positioned at the base of the chin and the forehead padding was adjusted to lie superior to the eyes. The facemask was centered by making sure the headform had a clear line of sight and that the mask was not tilted toward one side or the other. For the facemask that lacked a chin pad, the mask was positioned by centering the nose piece over the bridge of the nose and assuring a clear line of site. The head straps of each mask were adjusted to assure a snug fit around the FOCUS headform, preventing any mask from falling off the headform during testing.

A Phantom high speed camera (Miro Ic321s, Vision Research, Wayne, NJ) was positioned perpendicular to the balls trajectory to ensure correct impact location. The Phantom camera was set to a sampling rate of 1000 fps in order to capture the entirety of the impact. Prior to testing, the smallest distance between the interior side of the mask and the impacted facial bones were measured and recorded using a dial caliper. These measurements were used to analyze the effect of mask separation distance on facial fracture risk.

Data collected were processed according to SAE J211 and filtered using channel frequency class (CFC) 300 for the load cells and a CFC of 1000 for the linear accelerometers at the center of gravity of the headform.^7^ Peak resultant force for each facial bone was calculated for each test. Facial fracture risk was calculated using the nonparametric model developed by Cormier *et al*.^4^,^5^,^8^

Bare headform impacts were also conducted at 24.6 ± 0.51 m/s at the maxilla and zygoma locations as a reference for the forces experienced by a player not wearing a mask. The load cells of the FOCUS headform have a maximum load capacity of 4448.2 N for the facial bones and 1000.8 N for the eyes.^7^,^9^ It was determined that an impact of this severity would damage or break the instrumentation in the FOCUS, so as a result the tests were run using a 50^th^ percentile Hybrid III headform and the force was back calculated using the impulse-momentum theorem, Eq. (1).

1 $$m\Delta v = \int {Fdt}$$

In Eq. (1), *m* is the weight of the softball, ∆*v* represents the change in ball velocity from before to after the impact, *F* is the force experienced by the headform, and d*t* symbolizes the duration of the impact. The duration of the impact was acquired from the linear resultant acceleration pulse of the TDAS Slice Pro system and the change in velocity was obtained using the Phantom high speed camera. Five trials were conducted at each location and the average force was reported. For these tests, the 50th percentile Hybrid III headform was attached to the same 50th percentile Hybrid III neck and sliding table that was used for the FOCUS tests and the same ball model was utilized.

The Hybrid III headform was positioned to replicate the maxilla and zygoma impacts from the FOCUS tests. This meant that the headform was positioned 36.5 cm away from the end of the launcher in the *x* direction and tilted 10° toward the launcher. For the maxilla location the headform was rotated − 15° about the *z* axis and translated − 1.9 cm in the *y* direction and − 3.2 cm in the *z* direction from the reference location. For the zygoma location the headform was rotated − 55 about the *z* axis and translated + 3.2 cm in the *y* direction and − 1.8 cm in the *z* direction from the reference location. All transformations correspond with the head coordinate system for SAE J211 and the reference location for the Hybrid III is identified as the headform centered in front of the launcher with no rotations about any axis, and positioned so the middle of the muzzle is located at the tip of the nose. The Hybrid III headform was instrumented with three linear accelerometers at the center of gravity of the headform. The acceleration data were processed and collected according to SAE J211 and filtered using a CFC of 1000.

To determine if the mask material, or the distance between the mask and the impacted facial bone had an effect on mask performance for the specified impact locations, an ANCOVA was conducted using JMP Pro 13 (SAS, Cary, NC). The log transformation of the peak resultant force for each of the masks were used as the response variable in the ANCOVA because the risk values were zero heavy data and generated a non-normal distribution. The peak resultant force was identified as the peak resultant force of each facial bone for all the tests conducted, totaling 288 data points. Mask material and the distance between the mask and the impacted facial bone were used as predictors for each impact location in the analysis. A *p* value of ≤ 0.05 was considered significant. If either of these covariates are found significant in this analysis, the mask performance will be plotted by the covariates to allow for design recommendations to be made based on this specific test sequence.

## Results

Table 1 displays the average force and average non-parametric fracture risk of the nasal bone, the right maxilla, the right zygoma, and the right frontal bone for each mask at each impact location. The left maxilla, left zygoma, and left frontal bone were not included because they did not produce any fracture risk and the mandible was excluded because its risk values were relatively low.

**Table 1:** Average force (N) and average non-parametric fracture risk of the nasal, right zygoma, right maxilla, and right frontal bone for each mask at each impact location.

Mask	Impact location	Nasal		Right zygoma		Right maxilla		Right frontal	
		Force	Risk	Force	Risk	Force	Risk	Force	Risk
All-Star	M	291.70	0.21	38.41	0.00	917.80	0.34	562.73	0.00
	Z	89.37	0.00	1204.75	0.75	104.03	0.00	1005.25	0.05
Bangerz	M	413.55	0.28	102.72	0.00	2659.12	1.00	765.9	0.00
	Z	130.79	0.05	2952.91	1.00	308.47	0.00	2295.52	0.50
Champro	M	46.21	0.00	46.08	0.00	70.19	0.00	528.69	0.00
	Z	58.87	0.00	762.88	0.59	81.15	0.00	663.4	0.00
Defender Sports	M	282.75	0.21	470.19	0.00	441.27	0.06	311.95	0.00
	Z	155.69	0.09	2333.18	0.95	134.06	0.00	2748.77	0.64
Markwort Large	M	121.65	0.05	449.2	0.00	558.81	0.13	1535.16	0.25
	Z	110.98	0.05	2624.08	0.95	166.4	0.00	2020.77	0.50
Markwort Medium	M	91.43	0.00	193.8	0.00	742.80	0.26	1054.44	0.13
	Z	135.56	0.05	2784.31	0.95	168.18	0.00	2421.61	0.53
Rawlings	M	68.08	0.00	98.41	0.00	115.57	0.00	1690.50	0.30
	Z	141.33	0.05	2395.93	0.95	166.33	0.00	2017.52	0.50
Schutt Steel	M	77.34	0.00	44.86	0.00	97.03	0.00	893.1	0.05
	Z	83.37	0.00	788.37	0.59	101.22	0.00	1064.86	0.13
Schutt Titanium	M	87.23	0.00	50.52	0.00	124.54	0.00	1104.96	0.13
	Z	118.62	0.05	1357.32	0.87	125.01	0.00	1691.55	0.30

The Bangerz mask sustained the highest fracture risk value for the right maxilla during a maxilla impact (M) and zygoma impacts (Z) yielded the highest fracture risk value for the right zygoma across all masks. The left maxilla, left zygoma, and left frontal bones produced zero fracture risk and the mandible had relatively low fracture risks. Location M is a maxilla impact and location Z is a zygoma impact

**Figure 2:**
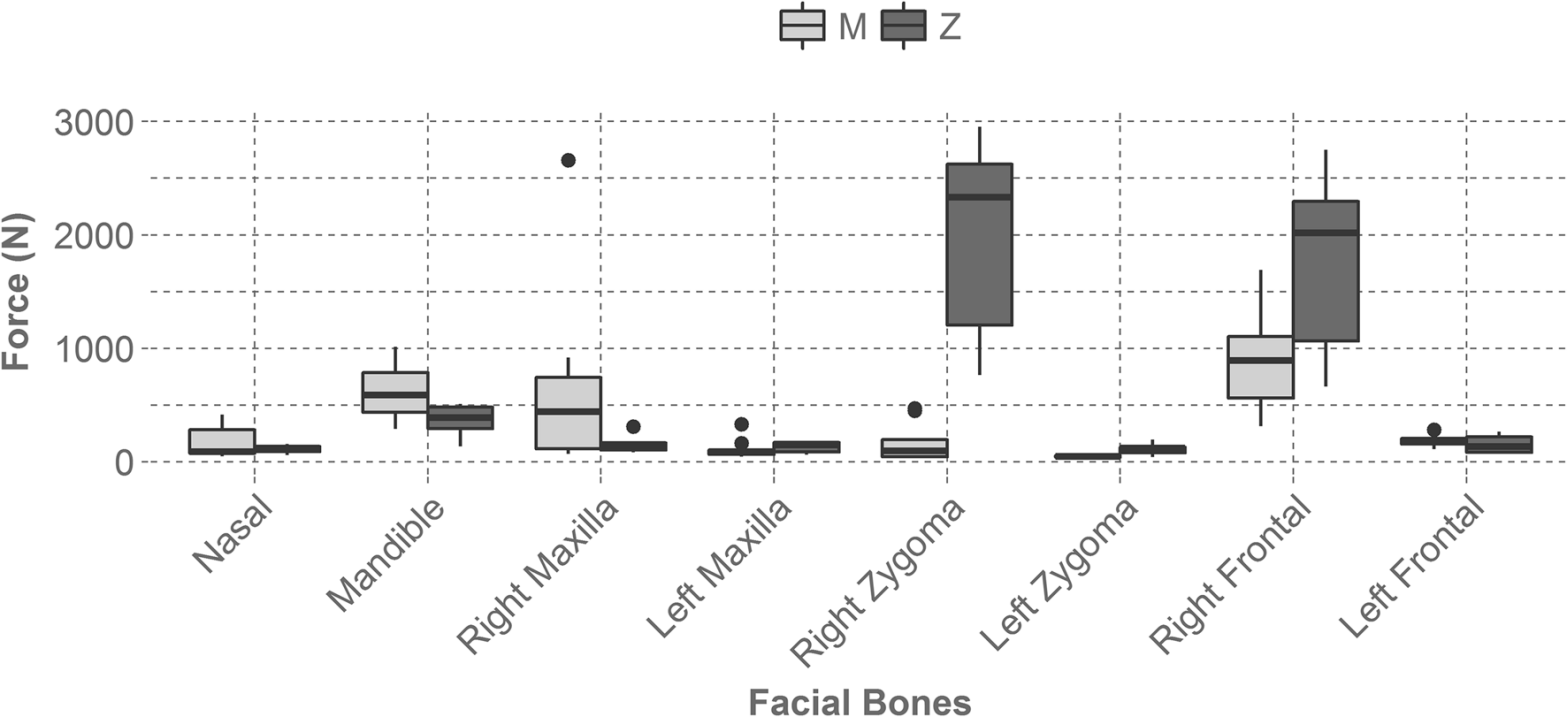
Illustrates the average force experienced by each facial bone across all mask types and location. The right zygoma and the right frontal bone experienced the largest forces during a zygoma impact (Z). The bones contralateral to the impact yielded relatively low forces. Location M is a right maxilla impact and location Z is a right zygoma impact.

Forces on facial bones varied by mask and location. The right zygoma and the right frontal bones yielded the highest forces during a zygoma impact (Z) for the tested masks (Fig. 2). However, there was a large range in forces within these impact configurations, indicating that some masks reduce force in these areas better than others. The forces tended to be greater in facial bones that were closer to the impact location than facial bones on the contralateral side of the impact.

**Figure 3:**
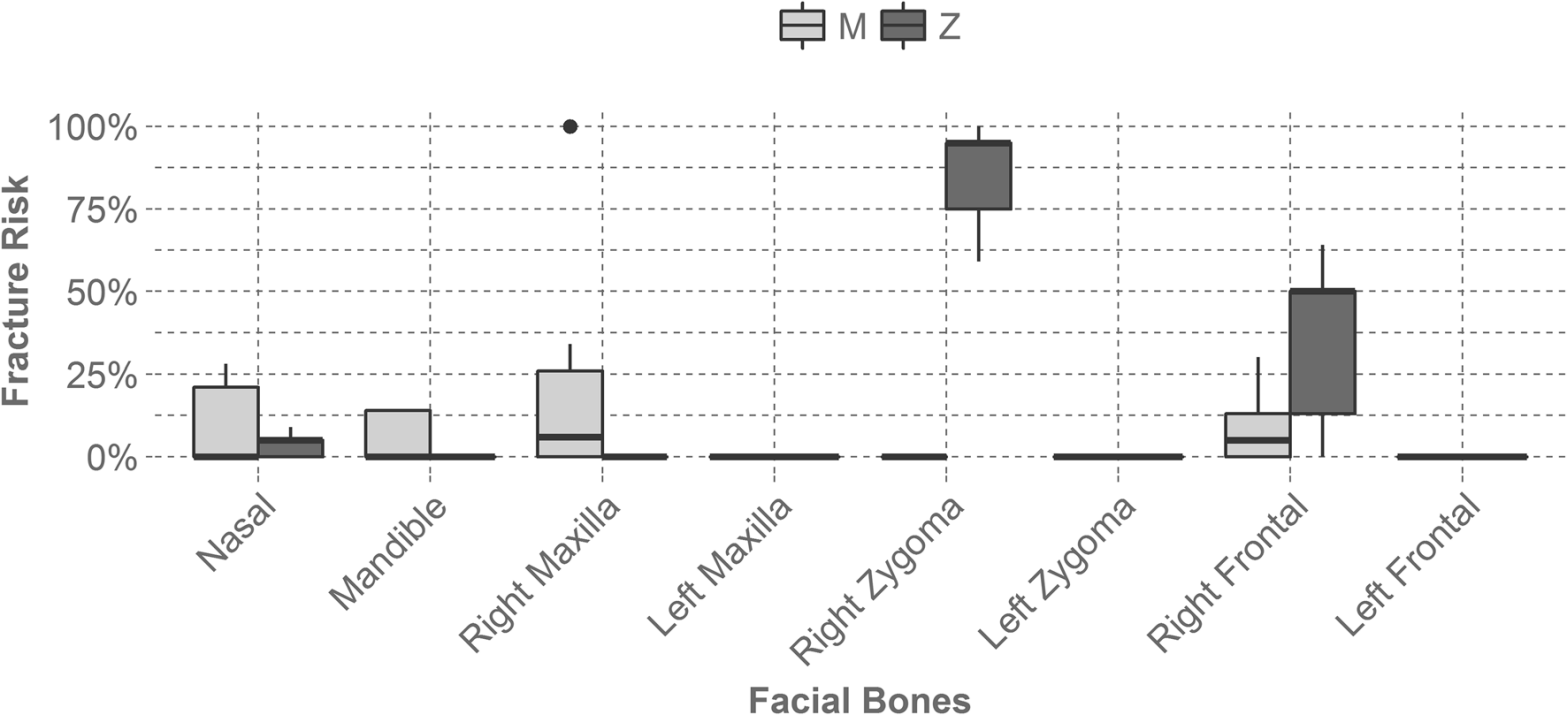
Displays the average non-parametric fracture risk seen by each facial bone across all mask types and by impact location. The right zygoma during a zygoma impact (Z) generated the highest fracture risk and facial bones contralateral to the impact produced no fracture risk. Location M is a right maxilla impact and location Z is a right zygoma impact.

**Figure 4:**
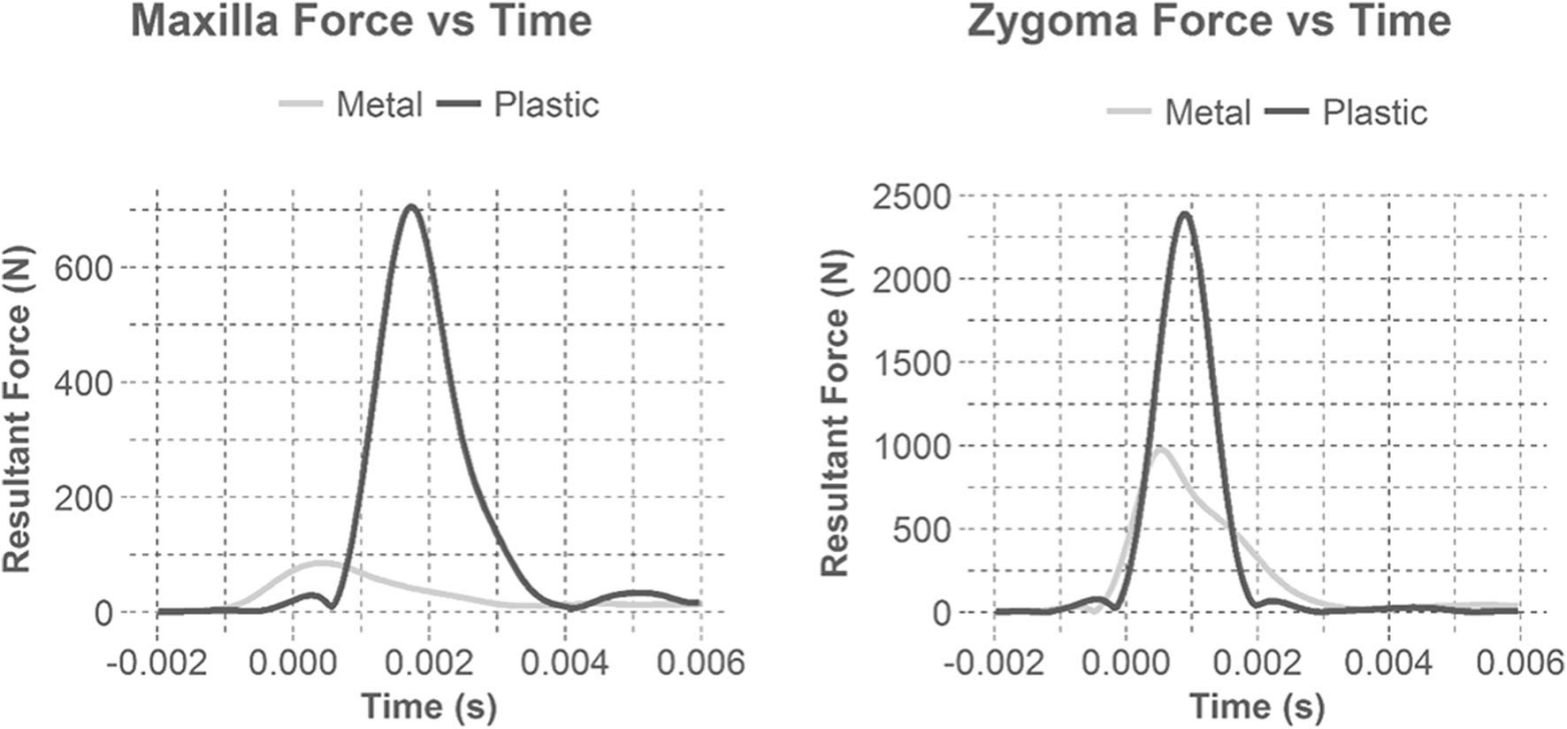
Illustrates example force time plots for metal and plastic masks during maxilla and zygoma impacts. Time zero was identified when the data acquisition was triggered (> 5 g in *x*-axis). Plastic masks yielded over double the force than metal masks and had steeper slopes indicating a higher loading rate. The higher loading rate is a result of the ball contacting the face during impact. For most cases, the masks rested on the zygoma, aligning the forces with the onset of impact. However, most masks did not initiate contact with the maxilla until later in the impact duration, resulting in the loading differences between the maxilla and the zygoma.

The average non-parametric fracture risk also varied by mask and location, but produced very similar trends to the average force (Fig. 3). The right zygoma bone during a zygoma impact (Z) generated the highest fracture risk. The facial bones on the contralateral side of the headform during this impact sustained zero fracture risk. Overall, the masks were successful at reducing facial forces and facial fracture risk. For comparison, if a player were to get hit in the maxilla without wearing a mask they would experience a force of 11,199 ± 651 N. Likewise, if a player were to get hit in the zygoma without wearing a mask they would experience a force of 10,826 ± 485 N. These forces were determined through the bare headform Hybrid III tests. The variation in facial force and fracture risk between the tested masks was significant and indicates that higher performing masks will reduce facial fracture risk, even at the more severe impact condition. Figure 4 displays exemplar force–time plots for metal and plastic masks at each impact location.

Each mask tested possessed its own unique design. Table 2 displays the differences by the mask material, weight, and the distances between the facemask and the impacted facial bone. Mask material encompassed polycarbonate, steel, and titanium, but was dichotomized so that all polycarbonate masks were classified as plastic and steel and titanium masks were classified as metal.

**Table 2:** Displays the material, the distance between the mask and the headform at the maxilla and zygoma, and the mass of each mask.

Mask	Material	Zygoma distance (mm)	Maxilla distance (mm)	Mass (g)
All-Star	Metal	23.30	46.62	430
Bangerz	Metal	16.35	26.47	140
Champro	Metal	29.50	45.00	370
Defender Sports	Plastic	19.77	47.29	185
Markwort Large	Plastic	24.97	61.74	215
Markwort Medium	Plastic	16.48	55.10	155
Rawlings	Metal	22.63	40.13	310
Schutt Steel	Metal	22.90	35.80	405
Schutt titanium	Metal	23.04	35.11	295

The material category “metal” includes both steel and titanium masks and the material category “plastic” represents polycarbonate masks. Plastic masks had greater distances between the mask and the maxilla when compared to metal masks

An ANCOVA between the log force, the mask material, the impact locations, and the distance between the mask and the impacted facial bone was conducted. The parameter estimates table, Table 3, was used to determine if distance and material were contributing factors to mask performance. Distance and material produced *p*-values of 0.0353 and 0.0021, showing that both distance and material effect mask performance. Impact location yielded an insignificant *p*-value (0.6931) showing that there was insufficient sample evidence to suggest that impact location had an effect on mask performance. There may be collinearity between impact location and distance that explains why impact location is not significant.

**Table 3:** Displays the parameter estimate table from the analysis conducted.

Term	Estimate	SE	*t* ratio	Prob > |*t*|
Intercept	2.68	0.15	18.00	< 0.0001
Material [metal]	− 0.11	0.03	− 3.11	0.002
Distance	− 0.01	0.00	− 2.12	0.04
Impact location [maxilla]	0.02	0.05	0.40	0.69

Since the material and the distance both had *p*-values less than 0.05, they were significant factors in a masks performance

**Figure 5:**
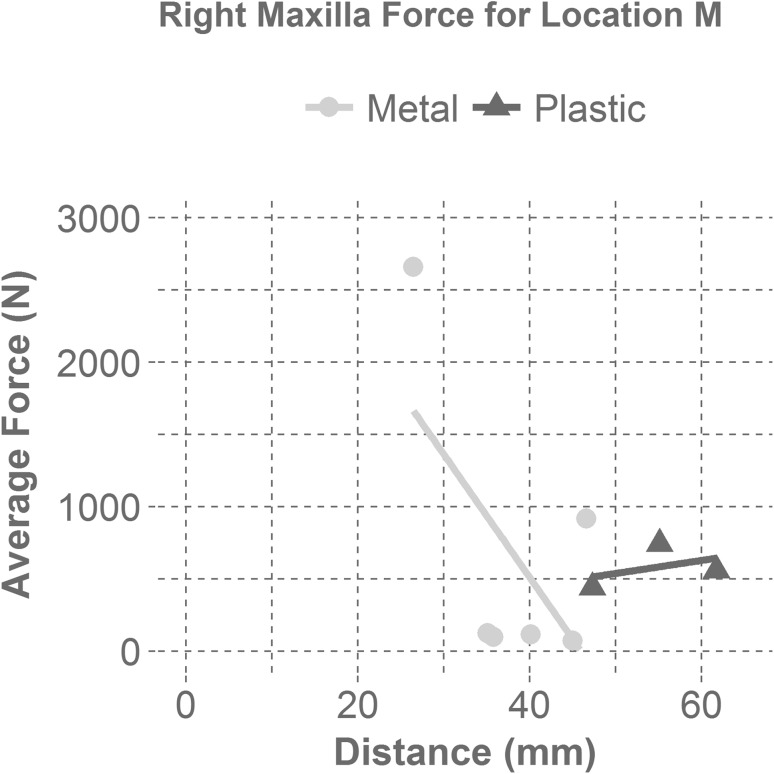
Shows the average right maxilla force as a function of the distance between the mask and the maxilla for each mask material at the maxilla impact location (M). The linear regression line for the metal masks displays a negative correlation with distance for a maxilla impact (M). The linear regression line for the plastic masks shows that there is little correlation between mask distance and average force for plastic masks in maxilla impacts (M).

**Figure 6:**
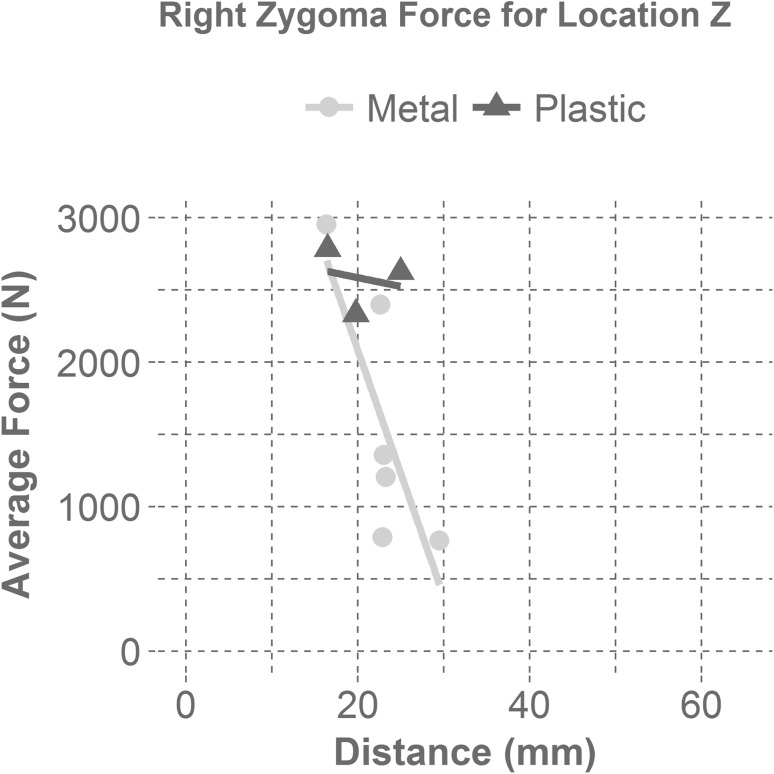
Displays the average right zygoma force as a function of the distance between the mask and the zygoma for each mask material at the zygoma impact location (Z). The linear regression line for the metal masks displays a steep negative correlation with distance for a zygoma impact (Z). The linear regression line for the plastic masks shows that there is little correlation between mask distance and average force for plastic masks in zygoma impacts (Z).

Figures 5 and 6 display the relationship between the average force and the distance between the mask and the impacted facial bone for each mask material (metal and plastic). Figure 5 presents the relationship between the average force and the distance by material for the right maxilla bone during a maxilla impact (M). Regardless of material, masks with a maxilla distance greater than 35 mm between the impacted facial bone and the interior of the mask resulted in an average force under 1000 N, which correlates to a fracture risk of approximately 38%.^8^ Figure 6 illustrates the relationship between the average force and the distance by material for the right zygoma bone during a zygoma impact (Z). Metal masks with a separation distance greater than 25 mm from the surface of the zygoma to the interior of the mask yielded average forces less than 1000 N, which correlates to a fracture risk of approximately 75%.^5^ More mask samples would provide a better understanding of the relationship between the average force and distance from the mask to the impacted facial bone for each mask material. Since force is a predictor of fracture risk, it is likely that fracture risk is affected by the same variables (material and distance from the mask to the impacted facial bone).

## Discussion

Evaluating the performance of infield masks using the FOCUS headform showed that masks do effectively reduce facial fracture risk. If a player is impacted at the maxilla while not wearing a mask they could experience a force of 11,199 ± 651 N. If impacted at the zygoma without a mask on a player could experience a force of 10,826 ± 485 N. The maximum force sustained during a maxilla impact (M) while wearing a mask was 2659 N (76% reduction in force) and the maximum force sustained during a zygoma impact (Z) while wearing a mask was 2953 N (73% reduction in force). Impacts to the zygoma location (Z) produced the highest forces and fracture risks. For zygoma impacts (Z), the right zygoma bone and the right frontal bone sustained the most severe forces and fracture risk values for the masks tested. However, these severe impact configurations yielded a range of forces between mask models that spanned from 762 N to 2953 N for the right zygoma bone during a zygoma impact (Z) and 663 N to 2749 N for the right frontal bone during a zygoma impact (Z). This corresponded to a fracture risk range from 59 to 100% for the right zygoma bone and a fracture risk range from 0 to 64% for the right frontal bone. These differences in facial force and fracture risk suggest that mask performance differs based on design and if the most effective mask is worn, based on the results seen from this series of tests, facial fracture risk can be reduced.

Looking into the design aspect of infield masks, it was found that mask material and the distance from the mask to the impacted facial bone were significant factors in mask performance. For all masks, the zygoma location (Z) had a smaller distance between the mask and the headform than the maxilla location (M), supporting the finding that a greater distance leads to a greater reduction of force. From analysis during a maxilla impact (M), if the distance was greater than 35 mm, the average force experienced was less than 1000 N (approximately a 35% fracture risk), regardless of mask material. However, a majority of the metal masks were able to reduce the average force more than plastic masks at this distance.^8^ During a right zygoma impact (Z), if the distance for a metal mask was greater than 25 mm, the average force was reduced to under 1000 N (approximately a 75% fracture risk) for our test configuration.^5^ Distance did not seem to effect the average force at the zygoma for plastic masks.

**Figure 7:**
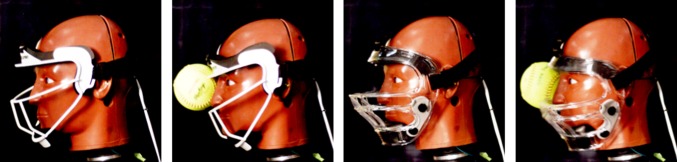
Shows the mask deformation comparison between metal and plastic for a maxilla impact (M). The left two pictures show the Champro mask (metal) before impact and during maximum ball intrusion. The right two pictures illustrate the Markwort Large mask (plastic) before impact and during maximum ball intrusion. The plastic mask deforms much more than the metal mask allowing the softball to contact the face.

**Figure 8:**
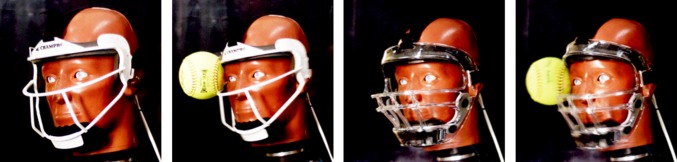
Displays the mask deformation comparison between metal (left) and plastic (right) for a zygoma impact (Z). The left two pictures show the Champro mask before impact and during maximum ball intrusion. The right two pictures illustrate the Markwort Large mask before impact and during maximum ball intrusion. The plastic mask deforms more than the metal mask allowing the softball to contact the zygoma.

It is believed that plastic masks performed worse than metal masks, even though their distances between the mask and the impacted facial bones were greater than or equal to metal masks, because the material properties of the plastic masks allowed significant intrusion. High speed footage depicted that as the softball engaged plastic masks, the masks deformed to the point where the ball contacted the headform (Figs. 7 and 8). If the ball is still able to contact the head through a mask, a higher amount of energy will be transferred into the head, instead of being dispersed to the mask, generating greater forces on facial bones. This is depicted in Fig. 4. The steeper slope of the plastic masks illustrate a higher loading rate than metal masks, which results from the ball contacting the face during impact. None of the masks broke upon impact from the ball, but there was permanent deformation of the metal masks at the impact site. The data suggest that a metal infield mask that has a clearance distance greater than 35 mm at the maxilla and greater than 25 mm at the zygoma best reduced facial fracture risk for these impact conditions.

In addition to evaluating infield masks ability to attenuate facial fracture risk, head acceleration data were collected to determine if wearing an infielder mask reduced head acceleration. During the bare Hybrid III impacts, it was found that the average linear resultant acceleration without wearing a mask for a maxilla impact was 226 ± 18 and 232 ± 14 g for a zygoma impact. These values serve as an estimate for a bare head impact to the FOCUS at the maxilla and zygoma respectively. Table 4 displays the average linear resultant acceleration for the maxilla and zygoma locations for each mask model. The min and max accelerations are presented instead of the standard deviation because only two trials were conducted for each mask at each location. The maximum acceleration seen at the maxilla location while wearing a mask was 168 g, which is a minimum reduction in acceleration of 26%. During the zygoma impacts, the All-Star, Champro, Rawlings, and Schutt Steel masks reduced acceleration by a minimum of 58%. However, the rest of the masks yielded acceleration values greater than or equal to the bare Hybrid III zygoma impact. These high acceleration values are likely due to variation in skin thickness between the two headforms at the zygoma location. The variation in skin thickness caused a shorter impact in the FOCUS compared to the Hybrid III, which supports the increased magnitudes seen during zygoma impacts on the FOCUS headform. These data suggest that some infield mask are capable of reducing linear head acceleration; however, in order to determine if infield masks effectively mitigate head injury risk rotational acceleration data is needed and tests should be run on the same headform to eliminate the effect structural differences between headforms have on the data.

**Table 4:** Displays the linear resultant head acceleration data for each infielder mask at each impact location with the min and max acceleration for each impact configuration.

Mask	Impact location	Average linear resultant (g)	Min/max
All-Star	Maxilla	88	81/96
	Zygoma	164	163/166
Bangerz	Maxilla	168	163/173
	Zygoma	449	442/457
Champro	Maxilla	44	39/49
	Zygoma	136	119/153
Defender Sports	Maxilla	61	57/66
	Zygoma	292	281/303
Markwort Large	Maxilla	140	122/158
	Zygoma	220	204/236
Markwort Medium	Maxilla	82	84/81
	Zygoma	216	205/228
Rawlings	Maxilla	92	87/96
	Zygoma	174	156/191
Schutt Steel	Maxilla	98	85/112
	Zygoma	144	115/172
Schutt Titanium	Maxilla	104	95/114
	Zygoma	260	249/272

The min and max were presented because there were only two trials for each impact configuration, so a standard deviation could not be calculated. The bare Hybrid III impacts resulted in an average linear resultant acceleration of 226 ± 18 g for a maxilla impact and 232 ± 14 g for a zygoma impact and serve as an estimate for a bare head impact to the FOCUS. All masks reduced head accelerations during maxilla impacts, however some mask yielded accelerations greater than or equal to the bare headform tests during zygoma impacts. This is likely due to variation in skin thickness between the two headforms at the zygoma location. These data suggest that some infield masks are capable of reducing head accelerations

While the study was able to evaluate the effect infielder masks had on facial fracture risk, there are a few limitations that should be acknowledged. First, force is only a correlate for predicting facial fracture. Knowing the force and the area engaged by the ball during impact would allow for pressure to be calculated, which is a better predictor of facial fracture.^4^ Furthermore, bare headform reference testing was not able to be conducted on the FOCUS headform for a direct comparison because of the possibility of breaking instrumentation. Another limitation is that the fracture risk calculated may not accurately represent the fracture risk of female high school softball players, since the model was developed using data from male cadavers ranging in age from 41 to age 94.^4^ Both the maxilla and zygoma bone strengths are not affected by age, but based on the loading conditions at each impact locations, the frontal bone during a lateral impact and the nasal bone during a direct impact may vary with age. For these specific scenarios the fracture risk is more conservative since bone strength decreases with age.^6^,^22^ In addition, the FOCUS headform is only made in one size, which may not accurately represent the head size of a female softball player. Furthermore, the sliding mass is modeled after the torso of a 50^th^ percentile male instead of a female high school softball player. Additionally, only two locations and one velocity were able to be evaluated because of limited resources. Since only a single severity was evaluated, some masks could perform better at higher or lower severities. Testing more locations and different severities would generate a better understanding of how these masks perform for all impact scenarios. Another limitation is that the head acceleration data were compared between two headforms that possessed structural differences. In order to completely understand the effect infield masks have on head injury risk, tests should be conducted on the same headform and both linear and rotational data should be collected. Finally, the number of trials and sample size were small. Conducting more trials would allow standard deviations to be calculated for each location. Adding other plastic and titanium masks to the sample would allow a better analysis of the relationship between the average force and the distance between the mask and the impacted facial bone and enable additional analyses to be done to determine if the type of metal has an effect on mask performance.

## Conclusion

Infielder masks are used to help reduce facial fracture risk in softball. To test if these masks can effectively reduce facial fracture risk, softballs were projected into the maxilla bone and zygoma bone of a FOCUS headform at 24.6 ± 0.51 m/s, representing the average batted ball speed for female high school softball players. Peak force was used to calculate facial fracture risk for each facial bone at both impact locations using a previously developed nonparametric risk model. It was found that infield masks do effectively reduce facial fracture risk. Mask material and the distance between the mask and the impacted facial bone were significant predictors of mask performance. Analysis of these factors justified that a metal mask with a distance of 35 mm or more above the maxilla bone and a distance of 25 mm or more above the zygoma bone is a good mask design for the loading conditions tested.

Although these masks did not eliminate the risk of facial fracture, they did reduce it. These data show that infield masks do effectively mitigate facial fracture risk and should be used to help prevent tragic injuries that could lead to facial reconstructive surgery, or in some cases death. Future studies can be conducted to better determine if head accelerations are reduced while wearing an infielder’s mask, which, when coupled with more trials and a greater sample size, can help to improve injury prevention in softball.
